# An observational study on the efficacy of targeted therapy for pulmonary sarcomatoid carcinoma

**DOI:** 10.1007/s12672-024-01046-5

**Published:** 2024-08-29

**Authors:** Takeshi Tsuda, Tomomi Ichikawa, Masahiro Matsumoto, Isami Mizusihima, Kenji Azechi, Naoki Takata, Nozomu Murayama, Kana Hayashi, Takahiro Hirai, Zenta Seto, Kotaro Tokui, Yasuaki Masaki, Chihiro Taka, Seisuke Okazawa, Kenta Kambara, Shingo Imanishi, Hirokazu Taniguchi, Toshiro Miwa, Ryuji Hayashi, Shoko Matsui, Minehiko Inomata

**Affiliations:** 1 Department of Respiratory Medicine, Toyama Prefectural Central Hospital, Toyama City, Japan; 2 Respiratory and Allergy Medicine, Toyama Red Cross Hospital, Toyama City, Japan; 3 First Department of Internal Medicine, Toyama University Hospital, Sugitani 2630, Toyama City, 930-0194 Japan; 4 Department of Medical Oncology, Toyama University Hospital, Toyama City, Japan

**Keywords:** Driver mutation, Pleomorphic carcinoma, Survival, Treatment

## Abstract

**Background:**

Pulmonary sarcomatoid carcinoma is a rare tumor that is resistant to cytotoxic agents. This observational study aimed to evaluate the detection rate of driver gene alteration and the efficacy of targeted therapy for pulmonary sarcomatoid carcinoma.

**Methods:**

We established a database of patients with pulmonary sarcomatoid carcinoma and their clinical information, including EGFR mutation, ALK fusion gene, ROS1 fusion gene, BRAF mutation, and MET exon 14 skipping mutation. The present study retrieved and analyzed the data of patients with pulmonary sarcomatoid carcinoma in whom driver gene alterations were evaluated, and the survival duration after the initiation of treatment with targeted therapy was examined.

**Results:**

A total of 44 patients were included in the present study. The EGFR mutation, ALK fusion gene, and MET exon 14 skipping mutation were detected in 2/43 patients (4.7%), 2/34 patients (5.9%), and 2/16 patients (12.5%), respectively. The ROS1 fusion gene (0/18 patients) and BRAF mutation (0/15 patients) were not detected. Female patients (P = 0.063, Fisher’s exact test) and patients without smoking history (P = 0.025, Fisher’s exact test) were the dominant groups in which any driver mutation was detected. Five patients with driver gene alterations were treated with targeted therapy. Progression-free survival (PFS) was 1.3 months and 1.6 months in 2 of the patients treated with gefitinib. Two patients with the ALK fusion gene showed 2.1 and 14.0 months of PFS from the initiation of treatment with crizotinib, and a patient with the MET exon 14 skipping mutation showed 9.7 months of PFS from the initiation of treatment with tepotinib.

**Conclusion:**

The EGFR mutation, ALK fusion gene, and MET exon 14 skipping mutation were detected in patients with pulmonary sarcomatoid carcinoma in clinical practice, and some patients achieved long survival times after receiving targeted therapy. Further investigation is necessary to evaluate the efficacy of targeted therapy for pulmonary sarcomatoid carcinoma.

## Introduction

Pulmonary sarcomatoid carcinoma accounts for less than 1% of all lung cancer and includes subtypes such as pleomorphic carcinoma, spindle cell carcinoma, giant cell carcinoma, carcinosarcoma, and pulmonary blastoma [1]. Pulmonary sarcomatoid carcinoma is resistant to cytotoxic agents, and survival after the initiation of treatment is generally short [2–4]. In addition, the efficacy of the combined therapy with cytotoxic agents plus vascular endothelial growth factor inhibitor is also limited, with progression-free survival (PFS) and overall survival (OS) estimated at 4.2 months and 11.2 months, respectively [5]. Although pulmonary sarcomatoid carcinoma is a very rare tumor, there is a crucial need to develop novel treatment strategies for this aggressive tumor [5].

In patients with non-small cell lung cancer (NSCLC), the identification of driver mutations and the development of targeted therapies have improved their prognosis [6–8]. The epidermal growth factor receptor (EGFR) gene is one of the most frequent driver oncogenes in NSCLC. Additionally, targeted therapies for the anaplastic lymphoma kinase (ALK) fusion gene, c-ros 1 (ROS1) fusion gene, BRAF mutation, mesenchymal-epithelial transition (MET) exon 14 skipping mutation, RET mutation, and KRAS mutation are available in NSCLC. The detection rates of these driver mutations are known to be different based on histological type, and they have been most extensively studied in lung adenocarcinoma [9]. For pulmonary sarcomatoid carcinoma, several gene alterations, including EGFR, ALK, MET, and KRAS, have been detected in previous studies [10–24], and some case reports are available regarding the clinical course of patients with pulmonary sarcomatoid carcinoma treated with targeted therapy [4, 18, 20, 25–30].

Given the poor prognosis of patients with pulmonary sarcomatoid carcinoma treated with cytotoxic agents, the efficacy of targeted therapy for pulmonary sarcomatoid carcinoma harboring driver gene alterations is an important issue. We conducted this observational study to accumulate more information on the detection rates of driver mutations and the efficacy of targeted therapy in patients with pulmonary sarcomatoid carcinoma.

## Methods

### Patient selection

We registered patients diagnosed as having pulmonary sarcomatoid carcinoma at Toyama University Hospital, Toyama Prefectural Central Hospital, or Toyama Red Cross Hospital and constructed a database using Research Electronic Data Capture (REDCap). Patients who were diagnosed based on surgical specimens (surgery, surgical biopsy, or autopsy) and those who were clinically diagnosed with pulmonary sarcomatoid carcinoma based on small specimens (transbronchial biopsy or needle biopsy) were enrolled. Clinical information, including driver mutations such as the EGFR mutation, ALK fusion gene, ROS1 fusion gene, BRAF mutation, and MET exon 14 skipping mutation, were recorded from medical charts. From the database, the data of patients in whom the driver mutation was evaluated were retrieved and analyzed in the present study.

This study was conducted in compliance with the Declaration of Helsinki and Ethical Guidelines for Medical and Biological Research Involving Human Subjects (Ministry of Health, Labour and Welfare, Japan). Prior to the start of the study, the study plan was approved by Ethics Committee, University of Toyama (Approved number: R2020099) and the ethical committees at each institution. Because the present study utilized existing information without any intervention or invasion, the need to obtain individual informed consent was waived, and research information was made available to research participants.

### Clinical information and treatment

Clinical information was collected, including driver mutation status such as EGFR, ALK, ROS1, BRAF, and MET. These driver mutations were evaluated in clinical practice by polymerase chain reaction (PCR), immunohistochemistry (IHC), and next-generation sequencing (NGS). Furthermore, data of the tumor programmed death ligand-1 tumor proportion score (PD-L1 TPS) evaluated in clinical practice using the 22C3 antibody were collected. Treatment choices were decided by attending physicians and conducted based on standard doses and schedules. Dose reduction or treatment discontinuation was decided by clinical judgment.

### Statistical analysis

PFS and OS after the initiation of the treatment with targeted therapy were calculated for the evaluation of treatment efficacy. PFS was calculated from the initiation day of the treatment with targeted therapy until the day at which disease progression was noted and censored at the last visit without disease progression. Disease progression was defined as clinically judged progression or progressive disease according to Response Evaluation Criteria in Solid Tumours version 1.1, whichever occurred first. OS was calculated from the initiation day of the treatment with targeted therapy until the day of death and censored at the last visit without death. Patient characteristics were compared using Fisher’s exact test. Statistical analysis was performed using JMP Pro version 17.0.0. (SAS, Cary, NC, USA).

## Results

### Patient characteristics

**Fig. 1:**
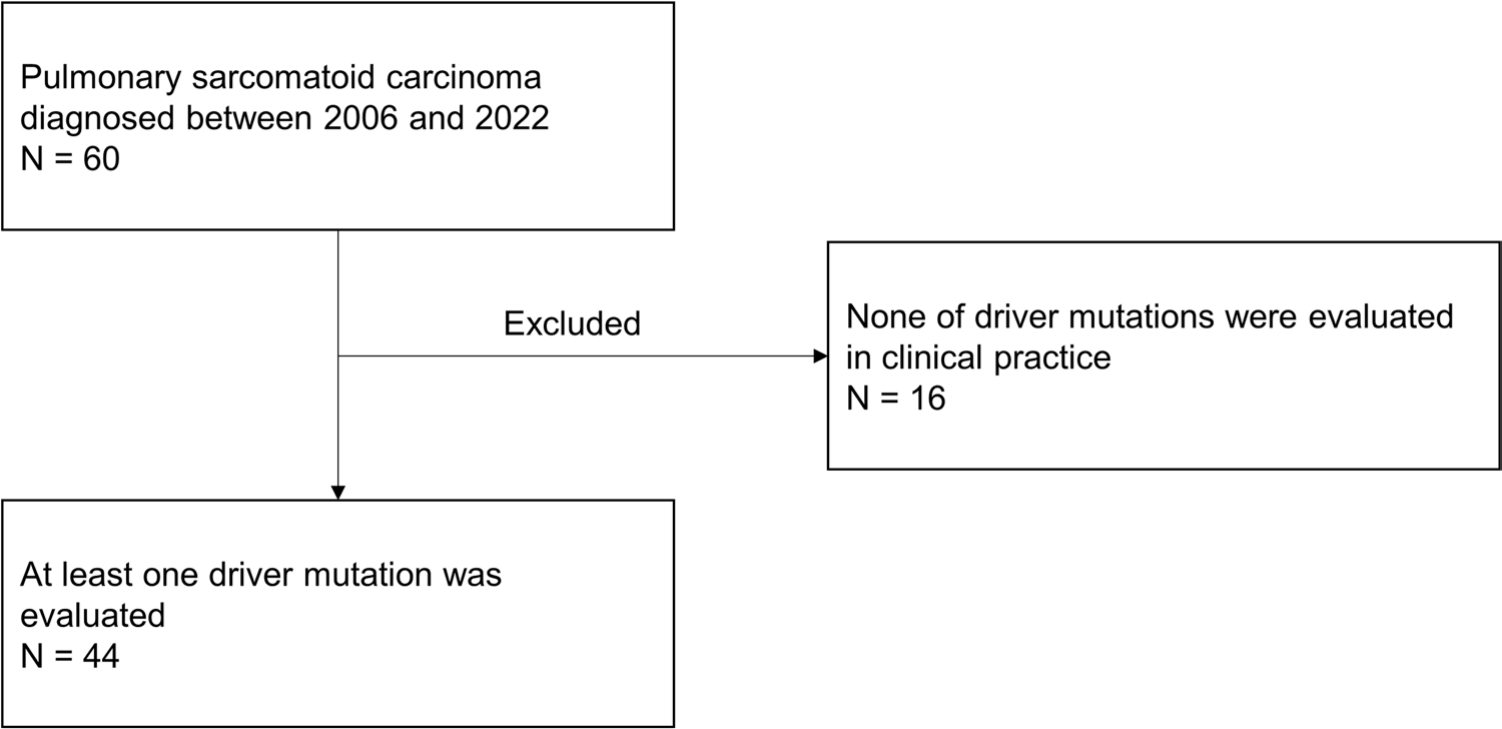
Patient selection

Data from 60 patients with pulmonary sarcomatoid carcinoma diagnosed between 2006 and 2022 were registered in the database. Among them, 44 patients in whom any driver mutation was evaluated were selected and analyzed in the present study (Fig. 1).

Patient characteristics are shown in Table 1. Male patients (36/44 patients, 81.8%) and patients with smoking history (38/44 patients, 86.4%) were dominant in the population. In 30 patients diagnosed by surgery, surgical biopsy, or autopsy, 25, 3, and 2 patients were diagnosed with pleomorphic carcinoma, carcinosarcoma, and spindle cell carcinoma, respectively. In 14 patients diagnosed using small specimens, including transbronchial biopsy or needle biopsy specimens, 10, 1, 1, and 1 patients were presumed to have pleomorphic carcinoma, carcinosarcoma, spindle cell carcinoma, and giant cell carcinoma, respectively. The remaining 1 patient was presumed to have sarcomatoid carcinoma due to the sarcomatoid component, but it did not reach to the specific diagnosis including pleomorphic carcinoma, spindle cell carcinoma, giant cell carcinoma, carcinosarcoma, pulmonary blastoma.

**Table 1:** Patient characteristics

		Whole population		Driver mutation detected		Driver mutation not detected		P
Sex	Male	36	81.8%	3	50.0%	33	86.8%	0.063
	Female	8	18.2%	3	50.0%	5	13.2%	
Age	< 70	24	54.5%	3	50.0%	21	55.3%	1.000
	≥ 70	20	45.5%	3	50.0%	17	44.7%	
Smoking history	Yes	38	86.4%	3	50.0%	35	92.1%	0.025
	No	6	13.6%	3	50.0%	3	7.9%	
Diagnostic procedure	Surgery	21	47.7%	4	66.7%	17	44.7%	0.946
	Surgical biopsy	6	13.6%	1	16.7%	5	13.2%	
	Autopsy	3	6.8%	0	0.0%	3	7.9%	
	TBB	8	18.2%	1	16.7%	7	18.4%	
	Needle biopsy	6	13.6%	0	0.0%	6	15.8%	

Age was recorded at the time of the first visit

*TBB* transbronchial biopsy

Driver mutations, including EGFR, ALK, ROS1, BRAF, and MET, were identified in 6 patients (13.6%). Female patients (3/6 patients, 50.0%, P = 0.063, Fisher’s exact test) and patients without smoking history (3/6 patients, 50.0%, P = 0.025, Fisher’s exact test) were dominant among the patients in whom driver mutations were detected.

### Detection rate of driver mutation

The detection rates of driver mutations are shown in Table 2. EGFR mutations were evaluated in most patients (43/44 patients), and 2 of the 43 patients (4.7%) showed an EGFR mutation of exon 19 deletion and exon 21 L858R by PCR testing. The ALK fusion gene was detected in 2/34 patients (5.9%) by IHC. Because the present study included patients previously diagnosed as having pulmonary sarcomatoid carcinoma, the ROS1 fusion gene, BRAF mutation, and MET exon 14 skipping mutation were not evaluated in a substantial proportion of patients. The ROS1 fusion gene (0/18 patients) and BRAF mutation (0/15 patients) were not detected. The MET exon 14 skipping mutation, however, was detected in 2/16 patients (12.5%) by NGS.

**Table 2:** Detection rates of driver mutations

	Number	Detection rate
EGFR		
Positive	2	4.7%
Negative	41	
Not evaluated	1	
ALK		
Positive	2	5.9%
Negative	32	
Not evaluated	10	
ROS1		
Positive	0	0%
Negative	18	
Not evaluated	26	
BRAF		
Positive	0	0%
Negative	15	
Not evaluated	29	
MET		
Positive	2	12.5%
Negative	14	
Not evaluated	28	
KRAS		
Positive	1	9.1%
Negative	10	
Not evaluated	33	

*ALK* anaplastic lymphoma kinase gene, *EGFR* epidermal growth factor receptor gene, *MET* mesenchymal-epithelial transition gene

In addition, we collected KRAS mutation information after the start of the study and found that KRAS G12C point mutation was detected in 1/11 patients (9.1%) by NGS.

### Survival

**Fig. 2:**
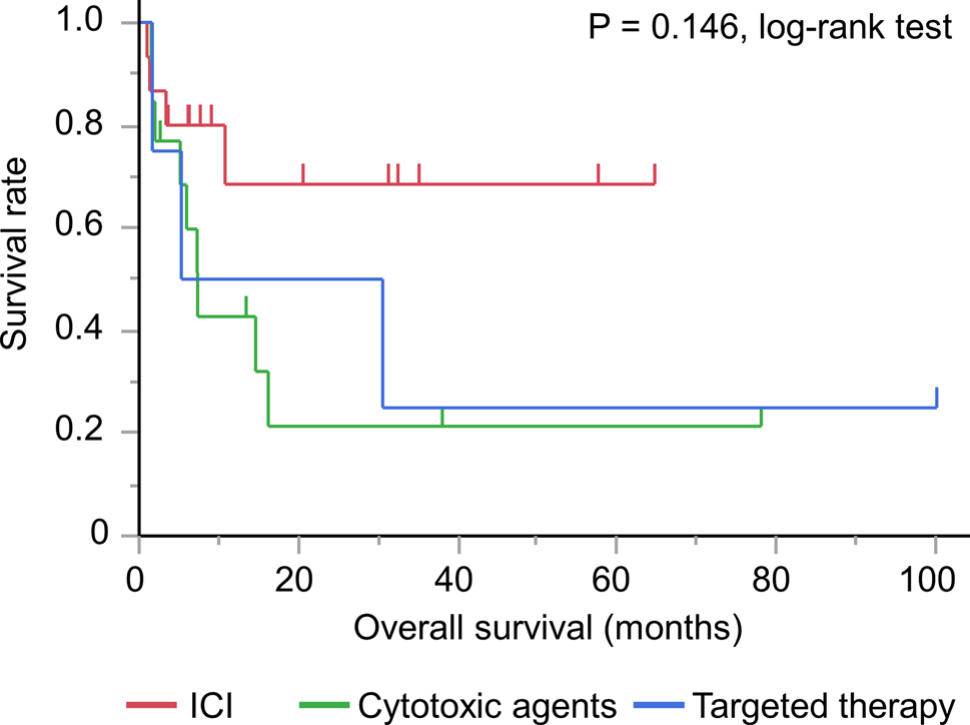
Kaplan-Meier curve for overall survival in patients with pulmonary sarcomatoid carcinoma receiving immune checkpoint inhibitor-containing therapy (n = 15), cytotoxic agents (n = 13), and targeted therapy (n = 4) as frontline treatment

Among 44 patients, 12 patients did not receive systemic therapy because they had poor performance status or there was no recurrence after surgery. In the remaining 32 patients, 15, 13, and 4 patients received immune checkpoint inhibitor-containing therapy, cytotoxic agents, and targeted therapy, respectively, as frontline treatment. One patient who received cytotoxic agents subsequently received targeted therapy (crizotinib) as second-line treatment. Median (95% confidential interval) overall survival from the start of frontline treatment was 16.2 (6.0-not estimated) months, not reached (3.4-not estimated), 7.3 (2.0–16.2) months, 17.9 (1.7-not estimated) months, in all patients, immune checkpoint inhibitor group, cytotoxic agent group, and targeted therapy group, respectively (Fig. 2).

The clinical courses of patients with positive driver mutations and who were treated with targeted therapy are shown in Table 3. A total of 5 patients, including 2 patients with the EGFR mutations 2 patients with the ALK fusion gene, and 1 patient with the MET exon 14 skipping mutation, were treated with targeted therapy. Additionally, another patient with the MET exon 14 skipping mutation did not receive targeted therapy because immune checkpoint inhibitor therapy provided an enduring response.

**Table 3:** Clinical courses of patients with pulmonary sarcomatoid carcinoma who were treated with targeted therapy

	Sex	Age	PS	Diagnosis	Smoking history	PD-L1 TPS	Driver gene	Test	TKI	Prior drug therapy	PFS (months)	OS (months)	Outcome
1	M	66	1	Surgery	Yes	unknown	EGFR	PCR	Gefitinib	No	1.3	5.3	Dead
2	F	71	NA	Surgery	Yes	95%	EGFR	PCR	Gefitinib	No	1.6	1.7	Dead
3	F	59	1	Surgical biopsy	No	unknown	ALK	IHC	Crizotinib	Yes	2.1	7.3	Dead
4	M	42	1	Surgery	No	10%	ALK	IHC	Crizotinib	No	14.0	100.2	Alive
5	F	83	1	Surgery	No	35%	MET	NGS	Tepotinib	Yes	9.7	21.3	Dead

Age and performance status were recorded at the initiation of targeted therapy

*ALK* anaplastic lymphoma kinase gene, *EGFR* epidermal growth factor receptor gene, *F* female, *IHC* immunohistochemistry, *M* male, *MET* mesenchymal-epithelial transition gene, *NA* not available, *NGS* next-generation sequencing, *OS* overall survival, *PCR* polymerase chain reaction, *PD-L1 TPS* programmed death ligand-1 tumor proportion score, *PFS* progression-free survival, *PS* performance status, *TKI* tyrosine kinase inhibitor

Both patients with the EGFR mutation who were treated with gefitinib showed less than 2 months of PFS (patient number 1 and 2). In 2 patients with the ALK fusion gene, 1 patient showed early progression after treatment with crizotinib (patient number 3). However, the other patient showed 14 months of PFS after the initiation of treatment with crizotinib. Furthermore, a long PFS of 83.3 months was observed after second-line treatment with alectinib (patient number 4). One patient with the MET exon 14 skipping mutation showed 9.7 months of PFS after the initiation of treatment with tepotinib. However, the treatment was discontinued owing to an adverse event (patient number 5).

## Discussion

In the present study, 6 of the 44 patients (13.6%) with pulmonary sarcomatoid carcinoma had driver mutations, including the EGFR mutation, ALK fusion gene, and MET exon14 skipping mutation. Among them, 5 patients were treated with targeted therapy, but the treatment efficacy was wide-ranging.

The present study revealed that the EGFR mutation, ALK fusion gene, and MET exon 14 skipping mutation were positive in 4.7%, 5.9%, and 12.5% of patients with pulmonary sarcomatoid carcinoma, respectively. The detection rates of these driver mutations were similar to those of previous reports in patients with pulmonary sarcomatoid carcinoma [10–24]. Furthermore, the present study showed that the majority of patients in whom any driver mutation was detected had no history of smoking. Chen et al. reported a similar result for the ALK fusion gene in pulmonary sarcomatoid carcinoma [18], and Forest et al. reported that adenocarcinomatous differentiation was associated with higher detection rates of driver mutations [14]. However, it was difficult to assess this in the present study because of insufficient histological information.

In the present study, PFS was less than 2 months in patients who were treated with gefitinib. Previous studies have reported on the clinical courses of 6 patients with EGFR-mutated pulmonary sarcomatoid carcinoma treated with EGFR-TKIs [4, 20, 25–28]. The shortest OS in these reports was 2 weeks [28]. Thus, the poor outcomes of EGFR mutant pulmonary sarcomatoid carcinoma presented in our study may not be surprising. However, there were several patients with 6 months of PFS or longer [4, 20, 26, 27], so it cannot be concluded that EGFR-TKIs are not effective for EGFR mutant pulmonary sarcomatoid carcinoma. In NSCLC, it has been reported that EGFR mutation status [31] and tumor PD-L1 expression [32–34] are associated with the efficacy of EGFR-TKIs, but there is not enough information regarding this association in pulmonary sarcomatoid carcinoma.

Patients who were treated with ALK or MET inhibitors showed relatively long PFS in the present study. It has been reported that patients with pulmonary sarcomatoid carcinoma harboring the ALK or ROS1 fusion gene showed a response to ALK and ROS1 inhibitors [18, 29, 30], and a phase II study of savolitinib, a MET inhibitor, showed a response rate of 49.2% in patients with pulmonary sarcomatoid carcinoma [35]. Although there is less information regarding the effectiveness of targeted therapy for pulmonary sarcomatoid carcinoma, given that some patients with pulmonary sarcomatoid carcinoma show long PFS and OS after the initiation of targeted therapy, it is important to consider and identify driver mutations in this population.

The present study had several limitations. First, a small sample size restricts the generalizability of the results. In addition, the retrospective nature of the study may have involved selective bias because patients were included in whom driver mutations were evaluated based on clinical judgment. Third, in the present study it may be difficult to generalize the detection rate of ROS1, BRAF, MET, and KRAS because a significant number of patients were not tested for these gene alterations. Finally, the diagnoses of pulmonary sarcomatoid carcinoma were based on surgical specimens, and lung cancer cases containing sarcomatoid components diagnosed using small specimens are defined as “non-small cell carcinoma with spindle cell and/or giant cell carcinoma” [1]. The present study included some patients who were clinically diagnosed as having pulmonary sarcomatoid carcinoma based on small biopsy specimens. However, because numerous advanced NSCLC cases are diagnosed using small specimens in clinical practice, the present study was considered to represent the real-world practice of pulmonary sarcomatoid carcinoma.

In summary, the present study showed that driver mutations, including EGFR gene mutations, ALK fusion genes, and MET exon14 skipping mutations, were detected in patients with pulmonary sarcomatoid carcinoma in clinical practice, and some patients achieved long survival times after receiving targeted therapy. This suggests that driver mutations must be considered in pulmonary sarcomatoid carcinoma.

## Data Availability

The datasets analyzed during the current study are available from the corresponding author on reasonable request.
